# Pairwise Attention: Leveraging Mass Differences to Enhance De Novo Sequencing of Mass Spectra

**DOI:** 10.1021/acs.jproteome.5c00063

**Published:** 2025-06-02

**Authors:** Joel Lapin, Alfred Nilsson, Mathias Wilhelm, Lukas Käll

**Affiliations:** † Computational Mass Spectrometry, TUM School of Life Sciences, Technical University of Munich, 85354 Freising, Germany; ‡ Science for Life Laboratory, KTH − Royal Institute of Technology, 171 65 Solna, Sweden; § Munich Data Science Institute, Technical University of Munich, 85748 Garching, Germany

**Keywords:** Attention, De novo sequencing, Transformers, MS2, Mass spectrometry, Proteomics

## Abstract

A fundamental challenge in mass spectrometry-based proteomics is determining which peptide generated a given MS2 spectrum. Peptide sequencing typically relies on matching spectra against a known sequence database, which in some applications is not available. Deep learning-based de novo sequencing can address this limitation by directly predicting peptide sequences from MS2 data. We have seen the application of the transformer architecture to de novo sequencing produce state-of-the-art results on the so-called nine-species benchmark. In this study, we propose an improved transformer encoder inspired by the heuristics used in the manual interpretation of spectra. We modify the attention mechanism with a learned bias based on pairwise mass differences, termed Pairwise Attention (PA). Adding PA improves average peptide precision at 100% coverage by 12.7% (5.9 percentage points) over our base transformer on the original nine-species benchmark. We have also achieved a 7.4% increase over the previously published model Casanovo. Our MS2 encoding strategy is largely orthogonal to other transformer-based models encoding MS2 spectra, enabling straightforward integration into existing deep-learning approaches. Our results show that integrating domain-specific knowledge into transformers boosts de novo sequencing performance.

## Graphical abstract

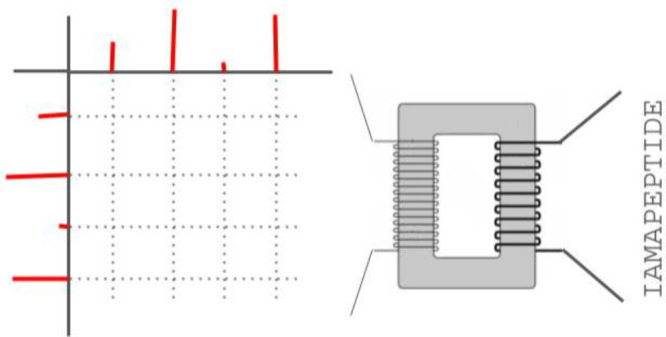

## Introduction

Identifying peptide sequences from tandem mass spectrometry (MS2) is currently dominated by sequence searching, where spectra will be matched to in silico digests of sequences from a sequence database.^1–5^ To obtain a high amount of identifications, one must choose a sequence database with a tenable search space size still containing sequences likely to be in the sample. Sequence searching inevitably has the weaknesses of bias and narrowness of the chosen sequence database, limiting the search only to those peptides the researcher believes will be present *a priori*.

De novo sequencing using deep learning^6–9^ (and traditional machine learning^10^) is an emerging approach that seeks to mitigate these weaknesses, wherein models can process the spectra and directly predict the peptide. These models, although not necessarily unbiased, can be trained on an expansive and diverse set of spectra, potentially overcoming the narrowness of sequence databases, provided that the model is reasonably accurate. Developed models have begun to be applied in areas where sequence searching is challenging. This can include applications where the search space is naturally large, such as immunopeptidomics,^11^ or the uncertainty around the content of the analyzed sample makes choosing a reference sequence database very difficult, as in antibody-sequencing,^12^ forensic samples^13^ and metaproteomics studies.^7,8^

The current state-of-the-art in de novo sequencing uses transformer models^14^ that frame de novo sequencing as a sequence-to-sequence translation problem.^15^ In these models, a list of spectral peaks is encoded by transformers into a latent representation using self-attention. The latent representation is further processed by a decoder model, which predicts a matching peptide’s amino acid sequence in an autoregressive manner, using cross-attention. Human experts often interpret MS2 spectra by seeking common patterns of backbone cleavages, e.g. b- and y-ions, for successive peaks that differ by the mass of single amino acids or small fragments of a peptide.^16^ Unlike human experts, deep learning models do not necessarily rely on predefined domain knowledge; instead, these models learn features by connecting the input to the target output using appropriate architectures, in our case the *m*/*z* and intensity peaks to a predicted amino acid sequence. A heavily parametrized model will automatically learn the features that best reduce the classification loss on the predicted sequence through gradient descent. However, such features are not easily decipherable, making these models practically uninterpretable, i.e., “black boxes”.

Despite the effectiveness of transformer models across a wide range of applications, leveraging domain knowledge can often improve performance beyond a direct naive implementation. In many applications, expert knowledge of the underlying problem is leveraged alongside the feature extraction capability of deep-learning modeling. In computer vision, convolutional neural networks^17^ incorporate an inductive bias by focusing on local receptive fields via learned filters and achieve state-of-the-art performance in many image tasks.^18–21^ An especially informative example of domain knowledge alongside deep learning is AlphaFold2 ^22^ in protein structure prediction. AlphaFold’s Evoformer is a deep learning module that processes evolutionary information from multiple-sequence alignments, including the modeled sequence and amino acid pairwise structural features of a protein structure. This is an architecture specifically designed for protein structure prediction. The peculiarities of the AlphaFold2 model, and how they relate to its specific field/problem of protein structure prediction, suggest that performance improvements can be achieved through careful consideration of domain-specific data and mechanisms underlying the problem.

Herein, we report the improvement of de novo sequencing by a transformer model incorporating features inspired by the traditional ways human experts would annotate an MS2 spectrum. We term our variation on the transformer as the Pairwise Attention model (PA), which concentrates on modifying the encoder half of the transformer to optimally process spectra into a latent space, used then for decoding the sequence. Specifically, for a spectrum of length N, we create an NxN set of features whose entries are the *m*/*z* differences of all pairs of peaks in the spectrum. Inspired by AlphaFold2’s pair representations of protein sequences, we feed this antisymmetric pairwise matrix into the transformer blocks as an attention bias. This augmentation to the transformer architecture is lightweight and programmatically simple to implement. We see large improvements over our own implementation of a transformer without such pairwise features, achieving a 14.2% (6.7 percentage point) increase in average peptide precision at 100% coverage over our base model and a 7.2% (3.6 percentage point increase) over Casanovo, when tested on the revised nine-species benchmark.

## Methods

Our model follows a standard encoder-decoder transformer architecture, but we modified the encoder’s self-attention mechanism to incorporate pairwise *m*/*z* differences as an additive bias.

### Peak Embeddings

The sequence of *N* peaks, each consisting of a mass and intensity *S* = {(*m*_*i*_, *I*_*i*_)}_*i* = 1_^*N*^, is fed into the transformer blocks per the standard spectrum encoding scheme, following PointNovo^6^ and Casanovo.^7^ Specifically, the *N* peaks of *m*/*z* and intensity values are expanded into Fourier features of dimension *r*_*m*_ and *r*_*I*_, respectively. Equations 1a and ([Disp-formula eq1b]) represent the processing of the original spectrum.

$$\phi_{i,p}^{\sin}=\sin\left(\frac{x_{i}}{\frac{\lambda_{\min}}{2\pi }{\frac{\lambda_{\max}}{\lambda_{\min}}}^{p/r}}\right)\;\mathrm{for}\;p\leq \frac{r}{2}$$ 1a

$$\phi_{i,p}^{\cos}=\sin\left(\frac{x_{i}}{\frac{\lambda_{\min}}{2\pi }{\frac{\lambda_{\max}}{\lambda_{\min}}}^{p/r}}\right)\;\mathrm{for}\;p>\frac{r}{2}$$ 1b

where *x*_*i*_ could either be the mass or the intensity of the *i*-th peak. Here, *p* indexes the Fourier feature dimension, and λ_max_ and λ_min_ are hyperparameters controlling the wavelength range.

The resulting Fourier features for each peak are concatenated along the feature dimension, producing an intermediate matrix of shape *N* × (*r*_*m*_ + *r*_*I*_). To transform this into the final input token matrix for the transformer, a linear projection is applied:

$$T=\mathrm{Linear}\left(\mathrm{Concat}\left(\phi_{i,p}^{\sin},\phi_{i,p}^{\cos}\right)\right),T\in R^{N\times d}$$ 2

where *d* is the desired input feature dimension for the transformer encoder.

### Pairwise Features

The 2D pairwise features (two sequence dimensions) are constructed by computing the pairwise differences between all pairs of peaks in the input spectrum. Specifically, for a sequence of *N* peaks, the pairwise *m*/*z* difference matrix $\Delta m\in R^{N\times N}$ is defined as

$$\Delta m_{i,j}=m_{i}-m_{j}$$ 3

where *m*_*i*_ and *m*_*j*_ are the *m*/*z* values of peaks *i* and *j*, respectively. To encode these pairwise differences, we expand each Δ*m*_*i*,*j*_ into Fourier features of dimension *r*_*pw′*_:

$$\Phi_{i,j,p}^{\sin}=\sin\left(\frac{\Delta m_{i,j}}{\left(\frac{\lambda_{\min}}{2\pi }\right){\left(\frac{\lambda_{\max}}{\lambda_{\min}}\right)}^{p/r_{pw\prime }}}\right)\;\mathrm{for}\;p\leq \frac{r_{pw\prime }}{2}$$ 4a

$$\Phi_{i,j,p}^{\cos}=\sin\left(\frac{\Delta m_{i,j}}{\left(\frac{\lambda_{\min}}{2\pi }\right){\left(\frac{\lambda_{\max}}{\lambda_{\min}}\right)}^{p/r_{pw\prime }}}\right)\;\mathrm{for}\;p>\frac{r_{pw\prime }}{2}$$ 4b

Let $\Phi \in R^{N\times N\times r_{pw\prime }}$ denote the resulting pairwise feature matrix.

### Pairwise Attention

Our mechanism for pairwise attention (Figure 1) borrows concepts from AlphaFold2’s ”MSA row-wise gated self-attention with pair bias” module, which must combine data of different modalities: a multiple-sequence alignment and pairwise amino acid encodings. Specifically, it feeds a pairwise representation of the sequence’s amino acids as a bias to the self-attention mechanism where keys, queries, and values originate from the multiple-sequence alignment. Our model mostly adheres to AlphaFold’s mechanism, but instead for Fourier features of the *m*/*z* sequence and for Fourier features of the pairwise *m*/*z* differences.

**Figure 1:**
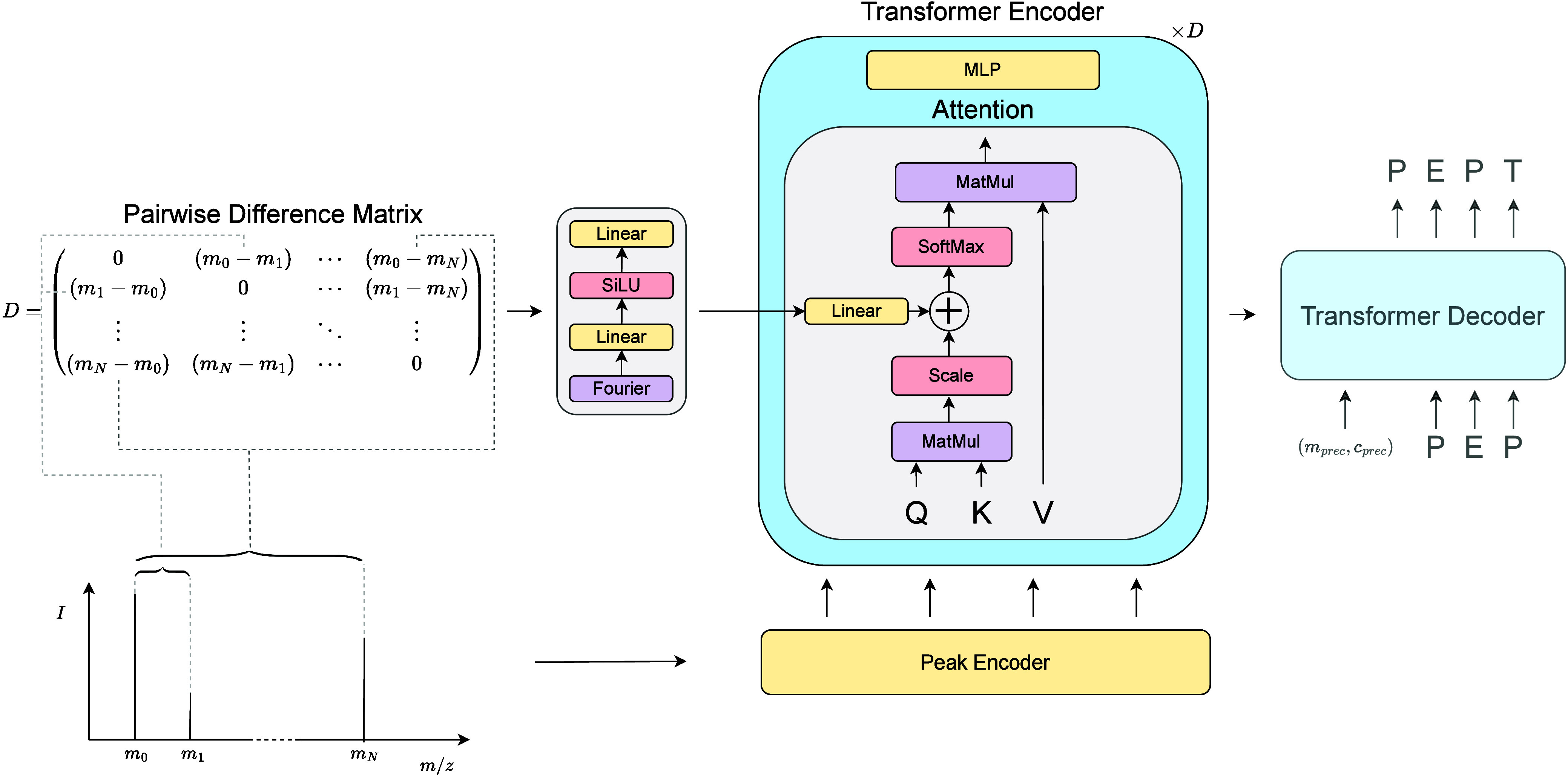
Architecture of the Pairwise Attention model, depicted through the self-attention mechanism of the Transformer encoder. The original mass spectrum in the lower left is turned into 1D features via the peak encoder, which concatenates Fourier features of the *m*/*z* and intensity dimensions, and further processed into 2D features by taking the pairwise differences of its *m*/*z* values. As the 1D features are processed by standard Transformer encoder modules, i.e. self-attention and multilayer perceptron (MLP) networks, the 2D features are fed into the self-attention module as a learnable bias before the softmax attention. This bias is fed into self-attention mechanisms throughout the depth of the encoder. The Transformer decoder is unaltered from the original implementation.^7,14^

Recall that transformer self-attention computes attention weights between all pairs of input positions. Given input embeddings $X\in R^{N\times d}$ for *N* peaks, we first compute queries *Q*, keys *K*, and values *V* for each attention head *h* and encoder layer *l*:

$$Q_{l,h}=X_{l}W_{Q}^{\left(l,h\right)}$$ 5

$$K_{l,h}=X_{l}W_{K}^{\left(l,h\right)}$$ 6

$$V_{l,h}=X_{l}W_{V}^{\left(l,h\right)}$$ 7

where $W_{Q}^{\left(l,h\right)},W_{K}^{\left(l,h\right)},W_{V}^{\left(l,h\right)}\in R^{d\times d_{h}}$ are learnable weight matrices, and *d*_*h*_ is the dimensionality per head, with *H* being the total number of heads.

The standard self-attention weights *A*_*l*,*h*_ for head *h* at layer *l* are then computed as

$$A_{l,h}=\mathrm{softmax}\left(\frac{Q_{l,h}K_{l,h}^{T}}{\sqrt{d_{h}}}\right),A_{l,h}\in R^{N\times N}$$ 8

To incorporate pairwise *m*/*z* differences, we define a learned function *f*_pw_(Φ), of the pairwise feature matrix (Equation 4a). This function maps the pairwise features to a latent space with dimensionality $\in R^{N\times N\times r_{pw}}$ . We then apply a *layer-specific* linear transformation *g* ^(*l*)^, which adapts the pairwise bias across the network depth. This results in the following attention activation map for each head *h* and layer *l*.

$$A_{l,h}^{\mathrm{pw}}=\mathrm{softmax}\left(\frac{Q_{l,h}K_{l,h}^{T}}{\sqrt{d_{h}}}+g^{\left(l\right)}\left(f_{h}^{\mathrm{pw}}\left(\Phi \right)\right)\right)$$ 9

where $A_{l,h}^{\mathrm{pw}}\in R^{N\times N}$ . For *f*_*h*_^pw^(Φ), we use two linear transformations with a SiLU activation in-between:

$$f_{h}^{\mathrm{pw}}\left(\Phi \right)=W_{2}^{\left(h\right)}\sigma \left(W_{1}^{\left(h\right)}\Phi +b_{1}^{\left(h\right)}\right)+b_{2}^{\left(h\right)}$$ 10

where (*W*_*i*_^(*h*)^, *b*_*i*_^(*h*)^) for *i* = 1, 2 are learnable parameters, shared across encoder layers, and σ is the SiLU activation function.

Each encoder attention module *l* applies its own linear transformation *g* ^(*l*)^ to the output of *f* ^pw^:

$$g^{\left(l\right)}\left(z\right)=U^{\left(l\right)}z+c^{\left(l\right)}$$ 11

where $U^{\left(l\right)}\in R^{r_{pw}\times h}$ and $c^{\left(l\right)}\in R^{h}$ are layer-specific parameters. This setup allows the network to adapt the pairwise bias across its depth.

#### Memory Footprint and Computational Cost

The pairwise features, each of which has dimension *r*_pw*′*_, are first linearly transformed to *r*_pw_ units through (*W*_1_, *b*_1_) of eq 10. When training in batches this operation creates a matrix of size batch size × *N* × *N* × *r*_pw_, which can result in substantial memory overhead when processing spectra with a large number of peaks. For this reason, it is important to select a conservative value for *r*_pw_, typically smaller than *r*_*m*_ or *r*_*I*_.

Inside the self-attention module, the pairwise features are linearly transformed to have the same number of channels as there are attention heads (*h*), and then added as the attention bias before taking the softmax, over the keys dimension. An illustration of the PA mechanism is depicted in Figure S1. By this mechanism, the pairwise features can exert great influence on the resultant attention map after the softmax is taken.

The total number of parameters added by the pairwise features is rather insignificant compared to the rest of the transformer. The first transformation of the pairwise Fourier features (*W*_1_, *b*_1_) adds *r*_pw_ *′*(2*r*_pw_ + 1) parameters, and (*W*_2_, *b*_2_) adds *r*_pw_(2*r*_pw_ + 1) parameters. *g* ^(*l*)^ is a linear transformation for each self-attention module along the depth of the encoder. Each attention transformation has size *h*(*r*_pw_ + 1) parameters. For a depth of D, this is a total parameter count for the entire network of (*r*_pw_ *′* + *r*_pw_)­(2*r*_pw_ + 1) + *Dh*(*r*_pw_ + 1). In this work, we use a model with *r*_pw_ *′* = 128, *r*_pw_ = 64, h = 8, D = 9 (see implementation details), which adds a total of 29,448 parameters, or about +0.1%, to the model. The VRAM memory cost increases from 9.3 GB (Base) to 17 GB (PA) for a single batch of 100 spectra.

### Data

We used two versions of the nine-species benchmark for comparison to other works. Our initial tests were on the original nine-species data set, referred to as nine-species V1. This data set is the smaller of the two, consisting of 1,526,282 total spectra. We downloaded Casanovo’s deposited preprocessed version of nine-species V1,^9^ published on Zenodo in 2022 (https://zenodo.org/records/6791263). The nine-species version 2 (V2) data set was researched by the Casanovo team to improve data quality and PSM confidence. This data set contained 2,844,842 total spectra. At the time of this writing, this data set was available in the Massive repository (ftp://massive.ucsd.edu/v05/MSV000090982/updates/2024-05-14_woutb_71950b89/peak/9speciesbenchmark/), where all relevant mgf files for each species can be downloaded and processed. For each version of the nine-species data set, we parsed all modified sequences to enumerate the tokens and establish the token dictionary that the model would use when run on that respective data set. The distribution of the number of spectra and peptides for each of the species in the set can be found in Table 1 To download the MassIVE-KB data set, we downloaded a metadata file provided from the MassIVE-KB Web site (https://massive.ucsd.edu/ProteoSAFe/static/massive-kb-libraries.jsp).^23^ The metadata file provides filenames and URL links for mzML files, from which we obtained the matching PSMs in the data set. We used 98.75% of the data for training, with a small development split of 1% for validation and 0.25% for testing. The true evaluation was done on each species of the nine-species data set.

**Table 1:** Number of Spectra and Unique Peptides in the Nine-Species Datasets V1 and V2

Species	Num. spectra V1	Num. pep. V1	Num. spectra V2	Num. pep. V2
*A. mellifera*	313,844	44,840	194,218	30,337
*B. subtilis*	291,769	34,258	1,358,337	66,455
*C. endoloripes*	150,117	16,959	82,290	10,884
*H. sapiens*	130,049	28,954	44,604	13,930
*M. mazei*	164,412	36,207	267,332	23,860
*M. musculus*	37,019	9,463	25,541	6,871
*S. lycopersicum*	290,000	92,934	178,413	62,467
*S. cerevisiae*	111,298	30,174	585,593	33,199
*V. mungo*	37,774	4,395	108,514	13,857
Total	1,526,282	298,184	2,844,842	261,860

In addition to the nine-species benchmark, we evaluated our models on an independent bacterial data set^24^ (PRIDE accession number PXD010613). This data set contains only variable modification of oxidized methionine and no fixed modifications. Because this data set is of bacterial origin, it is distinct from the MassIVE-KB training data, and the possibility of peptide leakage into the test set should be substantially lower than that for nine-species, providing a more rigorous test of model generalization.

### Adjustment of PEPMASS Annotation

During data preprocessing, we identified inconsistencies in the PEPMASS annotations within the mgf files of both nine-species V1 and V2 data sets. Specifically, the PEPMASS field erroneously contained the precursor *m*/*z* values instead of the precursor masses. In contrast, the MassIVE-KB data set’s PEPMASS field contained the precursor mass calculated as the product of the precursor *m*/*z* and charge, but without accounting for the mass of the protons (i.e., it did not subtract the proton masses associated with the charge state). It is worth acknowledging that there is no official standard for this field - the community does not agree upon the content, but according to MASCOT documentation (https://www.matrixscience.com/help/data_file_help.html), it should be populated with the peptide mass.

To ensure consistency across data sets, we addressed these discrepancies by adjusting the PEPMASS values in the nine-species V1 and V2 data sets. Specifically, we multiply the precursor *m*/*z* value in the PEPMASS field by the charge state and set this product as the new PEPMASS value:

Adj.PrecursorMass=Precursorm/z×Charge 12

This adjustment aligns the PEPMASS annotations in the nine-species data sets with those in the MassIVE-KB data set, where the PEPMASS field already contains this product.

While this correction does not account for the total proton mass associated with the charge state (i.e., it does not subtract Charge × *m*_*p*_, where *m*_*p*_ = 1.00727647 Da is the proton mass), incorporating the proton mass offset is unlikely to affect our machine learning model’s performance.

### Implementation Details

In order to have a fair comparison we largely adhered to Casanovo’s model configuration and hyperparameter settings for the PA model. We trained with 150 top intense peaks in a spectrum, and a maximum peptide length of 100. Spectral intensities were divided by the base peak, such that the base peak has *I*_*max*_ = 1 and all other peaks are scaled downward. The architectural parameters chosen for the spectral features from eq 1 were the following: *r*_*m*_ = 1024, *r*_*I*_ = 256, λ_max_^*m*^=10,000, λ_min_^*m*^=0.001, λ_max_^*I*^=1, and λ_min_^*I*^=1e-6. The parameters chosen for the pairwise features from eq 4b were *r*_*pw′*_ = 128, and the same frequencies as the spectral features. After concatenation of intensity and mass Fourier features, the tensor is linearly projected back to *r* = 512 units. The pairwise features are projected back to *r*_*pw*_ = 64; a smaller value than the 1d features was chosen to help stay within our GPU’s memory budget. Attention modules in the encoder were constructed with *d* = 64 query, key, and values units, *h* = 8 attention heads and 9 total attention blocks. The encoder was a custom implementation in Pytorch and had a total of 19.6 million parameters. The decoder had all the same hyperparameter settings as the encoder, but had 28.4 million parameters due to the extra cross attention modules. The decoder was implemented through the Depthcharge^25^ library (the same used by Casanovo) and altered to be compatible with our specific processing of the data. Dropout on both the encoder and decoder was set to 0.25.

We trained the PA model using the cross entropy loss function, implemented with Teacher forcing, where an attention mask prevents any token in the decoder from attending to future positions. Models were trained for 50 and 40 total epochs, for nine-species V1 and V2, respectively. A batch size of 100 was used. The learning rate was linearly warmed up to 2e-4 in 20,000 steps, and then held constant for the remainder of training. We used the Adam optimizer with default PyTorch parameters.

For the MassIVE-KB data set, instead of matching hyperparameters for comparison’s sake, we sought to train a more optimal realization of our model. We trained models that included 300 top intense peaks and tested on the nine-species V2 data set with a 5-beam beam search. Here, the learning rate was linearly warmed up to 2e-4 in 600,000 steps, and then held constant for the remainder of training. Models were trained for 10 epochs, after which the checkpoint with the best validation score was selected for final testing.

### Evaluation Metrics

All results for nine-species follow the procedure of training on 8 species and testing on the holdout ninth species. We report precision at 100% coverage (no confidence cutoff) at the peptide level. This is calculated as the number of predicted sequences that match the ground truth peptide divided by all peptides in the testing species’ data set, *N*_match_/*N*_total_. We follow the amino acid matching methodology first introduced by DeepNovo,^26^ wherein matched amino acids must be <0.1 Da and have a prefix or suffix mass that differs from the ground truth by <0.5 Da. To ensure that we do not simply optimize a specific random seed and have a robust result, all reported precision values for PA are the average of 3 random seeds, 0, 10, and 20. We only compare to Casanovo’s reported statistics, specifically those without a beam search. Casanovo has multiple published preprints with varying numbers for the nine-species benchmark; we specifically compared our results to their reported results in their most recently published article,^7^ provided in their Supporting Information.

For calculating the peptide confidence for the precision-coverage curves, we closely followed Casanovo’s peptide score metric. We take the mean softmax confidence of the predicted amino acids up to the stop token and set the confidence to – 1 for any predicted peptide’s mass that was more than 50 ppm off the precursor mass.

### Code Availability

All code is available for implementation and reproducibility of our work at https://github.com/statisticalbiotechnology/pairwise.

## Results

### Nine-Species Benchmark

We assess the PA model’s performance on the nine-species benchmark in two ways: 1) our results for our encoder with pairwise attention (PA), against the same model without pairwise attention, which we refer to as Base, and 2) our implementations against the reported results for Casanovo_bm_, which is their implementation without a beam search.^7^ Casanovo reported improvements over their top predecessors, PointNovo,^6^ DeepNovo,^9^ and Novor.^10^

The results and model comparisons for nine-species V1 are displayed in Figure 2 as peptide precision/coverage curves for all nine-species and the comparisons of precision at 100% coverage in Table 2 (individual precision/coverage curves for each species are displayed in Figure S3). Our PA model was run at 3 different seeds, 0, 10, and 20, and is reported with its standard deviation. When looking at average precision over all nine-species in Table 2, PA gives a boost of 11.3% (5.9 percentage points) peptide precision over the Base model. This is a substantial improvement given that PA adds only 29.5k more parameters, or approximately a 0.1 percentage points increase in the encoder size. Among the nine-species, the increases in performance range from 4 to 6 percentage points (*C. endoloripes*, *H. sapiens*, *M. mazei*, *M. musculus*, and *V. mungo*) to over 7 percentage points (*A. mellifera*, *B. subtilis*, *S. cerevisiae*, and *S. lycopersicum*).

**Figure 2:**
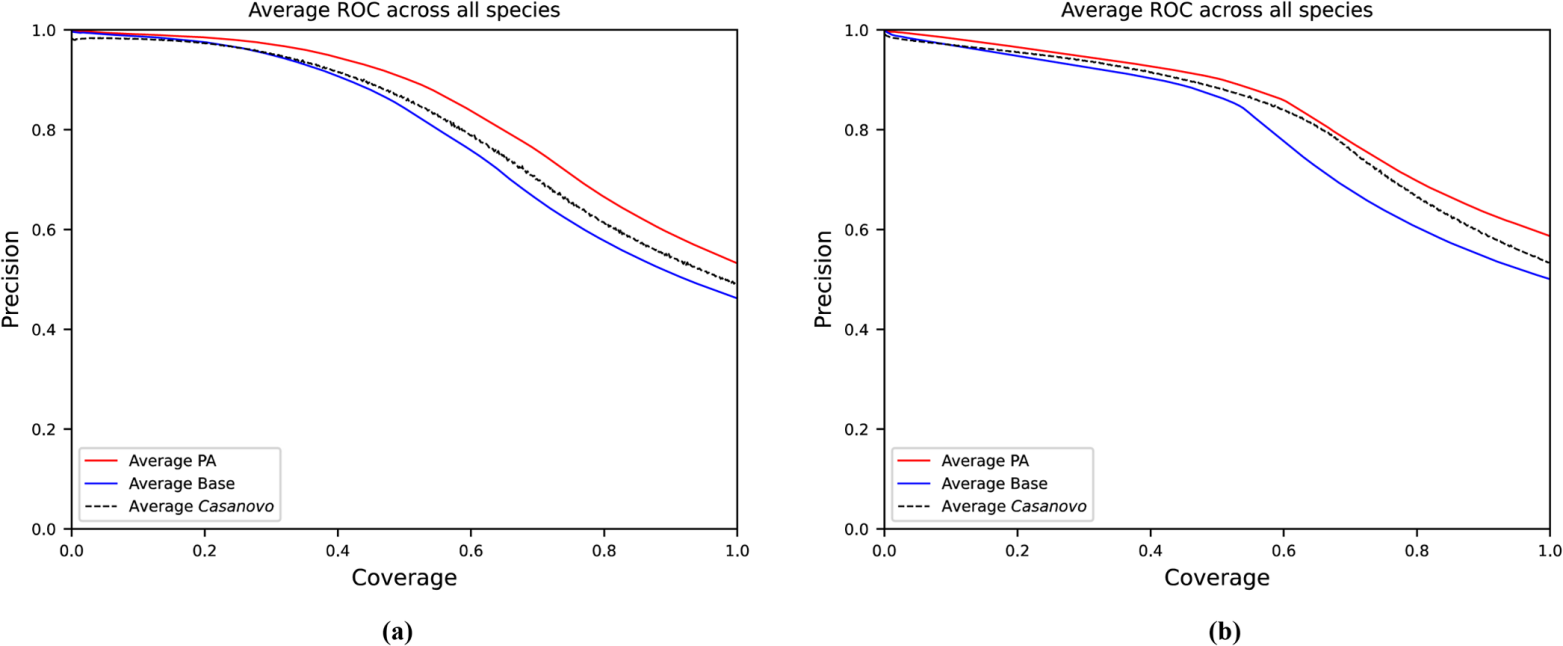
Precision-coverage curves for our PA and base models, and Casanovo’s reported BM model. Nine-species V1 is displayed in a) and V2 is displayed in b). For the PA model, only the best of the 3 seeds is plotted.

**Table 2:** Peptide Precision at 100% Coverage as Measured on the V1 and V2 of the Nine-Species Data Set^a^

	Nine-species V1			Nine-species V2		
Test Set Model	Base	PA	Casanovo	Base	PA	Casanovo
*A. ellifera*	0.390	0.463 (0.004)	0.433	0.390	0.446 (0.009)	**0.456**
*B. subtilis*	0.536	0.612 (0.009)	0.573	0.494	0.583 (0.006)	0.538
*C. endoloripes*	0.357	0.409 (0.002)	0.390	0.356	0.425 (0.006)	**0.468**
*H. sapiens*	0.340	0.391 (0.003)	0.383	0.451	0.521 (0.004)	**0.533**
*M. mazei*	0.503	0.554 (0.002)	0.515	0.509	0.579 (0.012)	0.529
*M. musculus*	0.433	0.472 (0.003)	0.431	0.410	0.435 (0.004)	0.395
*S. lycopersicum*	0.509	0.590 (0.008)	0.522	0.543	0.623 (0.007)	0.608
*S. cerevisiae*	0.537	0.612 (0.009)	0.580	0.558	0.631 (0.010)	0.561
*V. mungo*	0.570	0.625 (0.002)	0.552	0.525	0.598 (0.022)	0.428
Average	0.464	0.523 (0.004)	0.487	0.471	0.538 (0.009)	0.502

a Here, Base denotes our implementation of a standard transformer, i.e. without pairwise features. PA is our implementation of pairwise attention, and Casanovo is the reported numbers for Casanovo_bm_ in the original publication.^7^ For PA we report the average of 3 runs at seeds 0, 10, and 20, with standard deviation in parentheses. In addtion to precision at 100% coverage, Table S1 contains the average precision for all species and 9 species data sets.

When compared to the published results on the V1 data set, the PA model improves on Casanovo by 7.4% (3.6 percentage points). PA beats Casanovo for every species, with an exceptional increase of 6.8 percentage points for *S. lycopersicum*. It is important to make note of the disparity between our Base transformer implementation and Casanovo’s reported numbers, which is a considerable 2.3 percentage points on average. Nonoptimal hyperparameters may partly explain worse performance than Casanovo’s reported results, but is not sufficient to fully account for the overall difference in performance. It is possible that subtle different implementations of training/evaluation code and processing of data explain the inability to reproduce Casanovo’s original result, but were ultimately not identified in this study.

The nine-species V2 data set, constructed by the Casanovo authors, is reported to be higher confidence PSMs for each species. As of the time of this writing, Casanovo is the only other published model we are aware of that has reported results for this data set. When compared to the Base architecture, we see a very consistent increase in performance for PA overall, similar to the V1 data set. The average peptide precision increases by 14.2% (6.7 percentage points). A modest difference from V1 to V2 is not unexpected, as the updates to the nine-species data set significantly changed the size and likely also the quality of the data, but the persistence of the trends in improvement for all species shows that the advantage from pairwise attention is a robust and consistent result among our implementations.

When compared to Casanovo, again we see an improvement for our PA model by 7.2% (once again by 3.6 percentage points) in average peptide precision at 100% coverage. One important observation to make is how much more variability there is in the comparison of the two models than with nine-species V1. In nine-species V1, PA was consistently above or equal to Casanovo for all species. This is evident in the precision-coverage curve of Figure S3, where PA’s precision lies mostly above Casanovo’s precision for all confidences, for all V1 species. For nine-species V2 in Figure S4 Casanovo has equal or higher precision, over various confidence ranges, for *A. mellifera*, *C. endoloripes*, *H. sapiens*, and *S. lycopersicum*. When looking at performance at 100% confidence, in Table 2, the increases for our PA model range from 0 to 6 percentage points. Our model is performing worse for 3 species, *A. mellifera*, *C. endoloripes*, and *H. sapiens*; Casanovo exceptionally had 4.3 percentage points better peptide precision for *C. endoloripes*, whereas PA was better than Casanovo for this species by 1.9 percentage points on V1. For the rest of the species, our model was better, especially for *V. mungo*, which was 17 percentage points better than Casanovo while only 7 percentage points better on V1. Changes in the quality of the data notwithstanding, this result seems questionable because of the inconsistency with the results of V1 and the two models’ seeming unusual ability to specialize on specific train/test data splits. Furthermore, in contrast to Casanovo, the comparison between our Base and PA models is consistent across the two data sets. To reconcile this apparent incongruence in the results, it would be best to faithfully implement all models within the same platform/code base. We reserve this comparison for future work.

As a final demonstration on nine-species, we trained both our Pairwise Attention (PA) and Base models on the MassIVE-KB data set, using beam search and an increased maximum number of peaks per spectrum. The best-performing model was then evaluated on all species in the nine-species V2 data set; the results shown in Table 3 (individual precision/coverage curves for each species are displayed in Figure S5). Consistent with previously reported results for Casanovo,^7^ training on the MassIVE-KB data set led to substantial performance improvements compared to training on the smaller, lower-quality nine-species data set. The improvement of PA over Base, as shown in Table 3, is now 1.6% (2 percentage points on average) - narrower than when training on the V2 data set alone. This narrowing is expected with larger training data sets. However, the persistence of a clear gap between PA and Base, even with such an extensive data set (30 M spectra), underscores the significance of our results and suggests that the gains from our learned pairwise attention bias cannot easily be diminished by further scaling the training data.

**Table 3:** Performance after Training the Models on the MassIVE-KB Set^a^

	Nine-species V2		
Test Set Model	Base	PA	Casanovo
*A. mellifera*	0.618	0.640	**0.662**
*B. subtilis*	0.706	0.732	**0.778**
*C. endoloripes*	0.525	0.549	**0.656**
*H. sapiens*	0.737	**0.746**	0.740
*M. mazei*	0.700	**0.720**	0.710
*M. musculus*	0.563	**0.579**	0.552
*S. lycopersicum*	0.735	0.751	**0.799**
*S. cerevisiae*	0.765	0.784	**0.840**
*V. mungo*	0.753	**0.783**	0.762
Average	0.678	0.698	**0.722**

a Here we tested on each of the species in the nine-species dataset and report peptide precision at 100% coverage. The models tested are Base and PA, alongside Casanovo’s reported numbers.^7^

When compared to Casanovo, the PA model improved for *H. sapiens*, *M. mazei*, *M. musculus*, and *V. mungo*, and trails Casanovo for *A. mellifera*, *C. endoloripes*, *S. lycopersicum*, and *S. cerevisiae*. Notable among these latter species is the extent to which Casanova outperforms our PA model, by ∼ 5 percentage points for *S. lycopersicum* and *S. cerevisiae*, and exceptionally by 10.7 percentage points for *C. endoloripes*.

### External Bacterial Data Set

Although the nine-species benchmark is the most widely used data set for de novo sequencing studies, its limitations - including the age and quality of the spectra and instruments used for collection - raise concerns about its suitability for comparing deep learning models. While it remains useful for model prototyping, a performance comparison on a more contemporary data set is desirable. To address this, we evaluated our MassIVE-KB-trained models (without a beam search) on an independent bacterial data set and compared its performance to Casanovo’s own publicly available model checkpoint and code. Additionally, because the external bacterial data set is vastly different in origin from the MassIVE-KB training set, the likelihood of peptide leakage is minimal, making it a better test of generalization. Through this evaluation, we can allay concerns about the idiosyncrasies of the nine-species benchmark.

Table 4 reveals that the PA model lies 2.5 percentage points above Base, consistent with MassIVE-KB results in Table 3, but now both PA and Base considerably improve over Casanovo. On the bacterial data set PA is 8.5 percentage points (24% relative improvement) above Casanovo, in great contrast to the difference observed between the same model checkpoints evaluated on nine-species V2 and Casanovo’s reported numbers. Even more striking is that Base is now well above Casanovo’s performance, which was not the case in either Table 3 or the nine-species cross-validation in Table 2. It is important to emphasize that no hyperparameter tuning was done for training our models (or Casanovo) on this data set, and PA consistently improves over the Base model. As stated above, it is a future priority when comparing models to always run on the same data, in the same platforms, for fair comparison, as demonstrated here on the bacterial data set.

**Table 4:** Performance on an External Bacterial Dataset after Training the Models on the MassIVE-KB Set^a^

Model	Precision
Base	0.408
PA	**0.433**
Casanovo	0.348

a Here we tested on the bacterial test dataset and report peptide precision at 100% coverage. The models tested are Base, PA, and Casanovo’s own hosted MassIVE-KB model checkpoint and code.^7^

### Runtime cost

While the inclusion of pairwise features comes at a very light parameter cost, the increase in feature maps with sequence size (*N*, top peaks) that must be produced (and saved for backpropagation) by the model slows down forward and backward passes. For 3 runs of nine-species V1 with *V. mungo* as the holdout species, which was one of the species with the fewest spectra and thus one of the largest training sets, the average total training time was 90,177 s for 446,467 total training steps, or 4.95 training steps per second. For the Base encoder the average total training time was 62,291 s, 7.17 training steps per second, which is 31% faster. The cost in runtime must be weighed by both developers and users of the model, depending on application. All nine-species models were trained on a single A100 GPU, and the MassIVE-KB models on 16 A100 GPUs.

## Discussion

The results provided here show a substantial improvement in de novo sequencing performance when incorporating features that encode pairwise differences between all peaks in a spectrum. Specifically, our model with pairwise features demonstrates a substantial improvement over our baseline model without these features and a modest improvement over published results on the nine-species benchmark. The idea of adding such features was inspired by the intuition used when manually annotating spectra, where one often identifies sequences from pairs of peaks that differ by the masses of modified and unmodified amino acids.

It can be argued that handcrafted features or inductive biases are unnecessary, as transformers have the capacity to learn higher-ordered features such as the pairwise differences and their correspondence to different fragments on their own. We see evidence suggesting that during training, the Base model may be learning representations of pairwise features, or approximations of them. For example, the pairwise model consistently shows a faster decrease in training loss and improved validation peptide precision compared to the base model (Figure S2), although the performance gap narrows over time. Nevertheless, after both models converge to their best validation scores, the pairwise model continues to outperform the Base model in the validation metrics and ultimate test performance.

This suggests that the transformer’s capacity to solve de novo sequencing may be limited when relying on randomly initialized models optimized by gradient descent. Handcrafted features, such as the pairwise distances, can direct the model to converge faster. It is also possible that the inclusion of high order features as input from the start of training might relieve the model to learn such features from scratch allowing the procedure to focus on the learning of even higher ordered features. Our results show that feature engineering, which traditionally has been essential for machine learning techniques such as decision trees and support vector machines, can still play a valuable role in deep learning alongside raw inputs.

The PA model was better than the Base model for both the nine-species data set V1 and V2, but then only slightly better for each respective architecture trained on MassIVE-KB. It is possible that pairwise attention is most beneficial when training data is limited, since MassIVE-KB is more than 10 times the size of nine-species V2. Other possibilities are that PA is most helpful when data quality is low and that MassIVE-KB contains higher-quality PSMs than the nine-species data set. Further testing is needed to elucidate the advantages of PA, and in what situations they may be marginalized.

It should be noted that our model has the same transformer implementation of the peptide decoder as Casanovo (Depthcharge), with architectural differences only in the encoder. As our Base encoder is a custom implementation of the standard transformer encoder, it should be very close in implementation to Casanovo’s encoder, and thus the overall models are nearly identical. The disparity in the performance between our Base model and Casanovo’s reported result on nine-species, despite their similarities, cannot fully be explained in this work. One possible factor is that we were unable to identify the ideal hyperparameters for the model, due to the computational demands of the nine-species benchmark, which made it difficult to exhaustively optimize all settings. Furthermore, our focus was on evaluating pairwise features’ contribution to performance in isolation, thus we largely copied the hyperparameters chosen by Casanovo to make a fair comparison.

Uncertainty in the data and hyperparameters notwithstanding, we tested all models, including Casanovo’s provided checkpoint, on an independent bacterial data set, applying our same model checkpoints as used in Table 3. With this data set we found consistency between Base and PA, and now a considerable improvement over Casanovo. This evaluation further supports the merits of using pairwise features as input to the attention mechanism. Since pairwise features integrate seamlessly with transformer-based de novo models, with consistently higher performance in both our nine-species ablation studies and on our independent bacterial data set, we believe this is an important contribution to the field as de novo sequencing becomes mainstream alongside traditional database searches.

## Supplementary Material



## Notes

Table S1. Average peptide precision (AUC) on V1 and V2 of the nine-species data set. Figure S1. Architectural schematic of the Pairwise Attention encoder. Figure S2. Training loss vs epoch on the nine-species V1 data set for base and pairwise models. Figure S3. Peptide precision vs coverage on the nine-species V1 data set for each species (held-out evaluation). Figure S4. Peptide precision vs coverage on the nine-species V2 data set for each species (held-out evaluation). Figure S5. Peptide precision vs coverage on the nine-species V2 data set after training on the MassIVE-KB data set (pretrained evaluation) (PDF)
