# Supplementary material for: Pairwise Attention: Leveraging Mass Differences to Enhance De Novo Sequencing of Mass Spectra
Source: J Proteome Res. 2025 Jun 2;24(7):3722–30. doi: 10.1021/acs.jproteome.5c00063 (PMC12235698; doi:10.1021/acs.jproteome.5c00063)
Supplement: Supplementary file 1 [file pr5c00063_si_001.pdf]

Supplement to "Pairwise Attention: Leveraging Mass Differences to Enhance De Novo Sequencing of Mass Spectra"

Joel Lapin [#1] **Alfred Nilsson** [#2] **Mathias Wilhelm**[*13] **Lukas Käll**[*2]

[1] Computational Mass Spectrometry, TUM School of Life Sciences, Technical University of Munich, Freising, Germany
[2] Science for Life Laboratory, KTH – Royal Institute of Technology, Stockholm, Sweden
[3] Munich Data Science Institute, Technical University of Munich, Garching, Germany

[#] Equal contribution [*] Corresponding Authors
mathias.wilhelm@tum.de, lukas.kall@scilifelab.se

Supporting information

The following supporting information is available free of charge at the ACS website http://pubs.acs.org and in the file `supplementary_information.pdf`:

- **Table S1.** Average peptide precision (AUC) on V1 and V2 of the nine-species dataset.
- **Figure S1.** Architectural schematic of the Pairwise Attention encoder:
- **Figure S2.** Training loss vs. epoch on the nine-species V1 dataset for base and pairwise models.
- **Figure S3.** Peptide precision vs. coverage on the nine-species V1 dataset for each species (held-out evaluation).
- **Figure S4.** Peptide precision vs. coverage on the nine-species V2 dataset for each species (held-out evaluation).
- **Figure S5.** Peptide precision vs. coverage on the nine-species V2 dataset after training on the MassIVE-KB dataset (pretrained evaluation).

Table S1. **Average peptide precision (AUC) on V1 and V2 of the nine-species dataset**

TEST SET	NINE-SPECIES V1			NINE-SPECIES V2		
MODEL	BASE	PA	CASANOVO	BASE	PA	CASANOVO
Apis Mellifera	0.735	**0.802**	0.767	0.699	0.759	**0.768**
Bacillus Subtilis	0.852	**0.899**	0.866	0.799	**0.861**	0.832
Candidatus Endoloripes	0.700	**0.750**	0.720	0.671	0.742	**0.772**
Homo Sapiens	0.681	**0.738**	0.722	0.754	0.817	**0.831**
Methanosarcina Mazei	0.852	**0.899**	0.866	0.799	**0.861**	0.832
Mus Musculus	0.770	**0.806**	0.766	0.700	**0.747**	0.721
Solanum Lycopersicum	0.833	**0.890**	0.837	0.831	0.881	**0.883**
Saccharomyces Cerevisiae	0.855	**0.889**	0.871	0.833	**0.879**	0.850
Vigna Mungo	0.868	**0.892**	0.849	0.811	**0.870**	0.746
AVERAGE	0.791	**0.836**	0.804	0.767	**0.824**	0.804

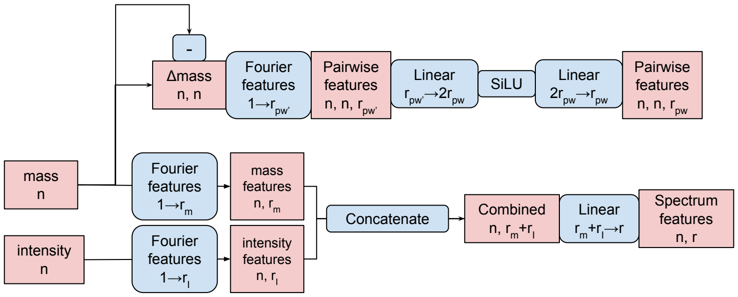

(a)

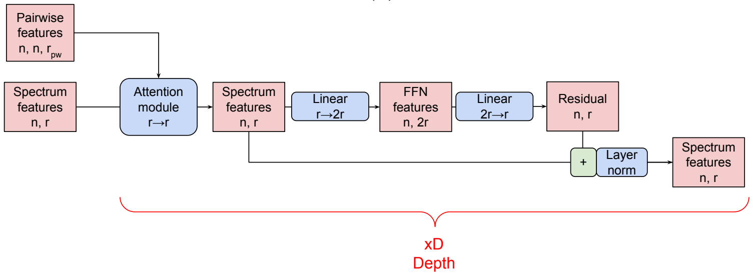

(b)

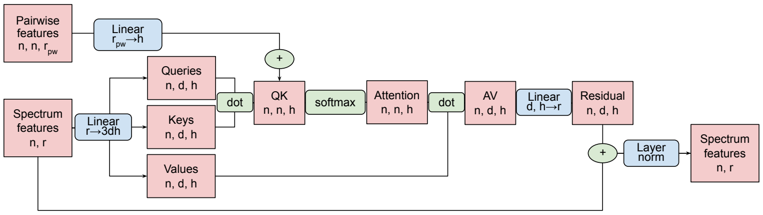

(c)

Figure S1. Schematic depicting the architectural integration of pairwise features into the Pairwise Attention model's encoder. In a) the preprocessing of mass and intensity vectors produces both spectral and pairwise features, which are then input into the main transformer blocks. In b) the repetitive unit of the encoder is outlined. Spectral features are fed into the first block of the encoder, while pairwise features are fed into every attention module throughout the depth of the encoder. The mechanism of communication between the spectral features and pairwise features is depicted in c). Besides the pairwise features, this is a standard self-attention module, but with them, there is an extra linear transformation to the number of heads and an addition operation as a bias before the softmax. Not pictured is the transformer decoder, which for this project is the native pytorch layer implementation.

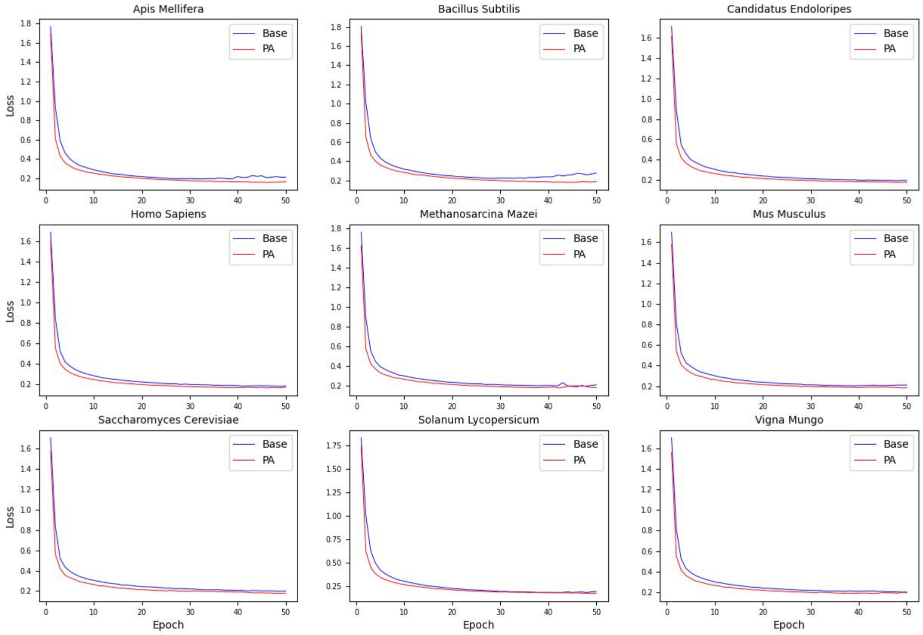

Figure S2. Training loss as a function of epoch for V1 of the nine-species dataset for our base and pairwise models. Training loss was averaged over every epoch.

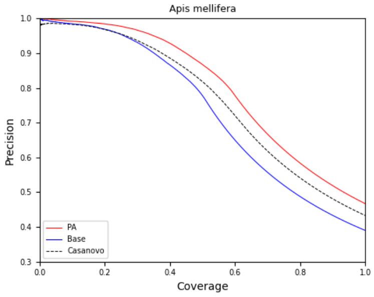

(a) *Apis mellifera*

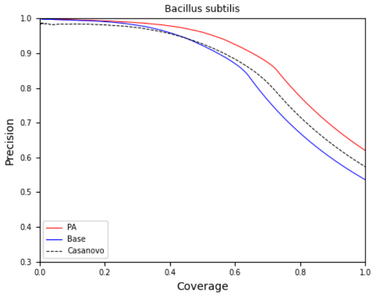

(b) *Bacillus subtilis*

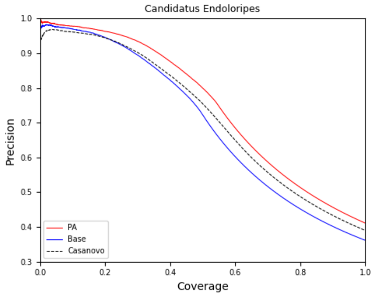

(c) *Candidatus endoloripes*

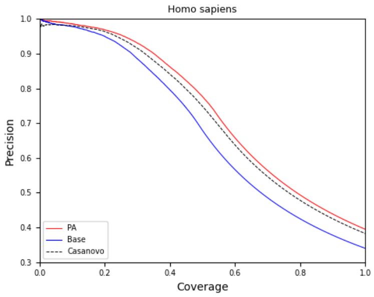

(d) *Homo sapiens*

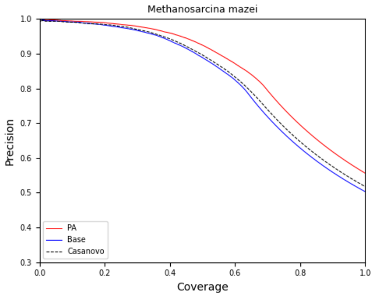

(e) *Methanosarcina mazei*

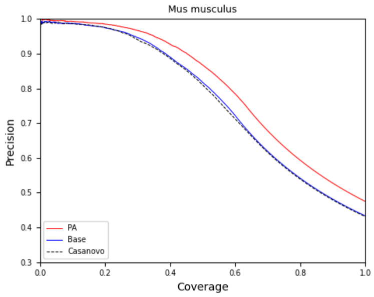

(f) *Mus musculus*

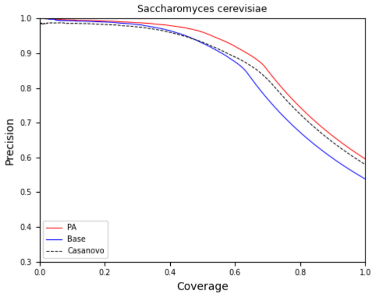

(g) *Saccharomyces cerevisiae*

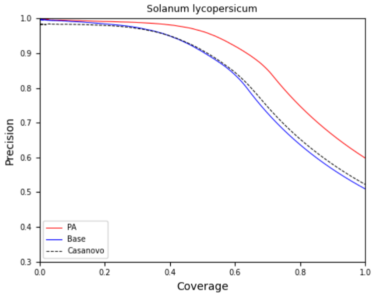

(h) *Solanum lycopersicum*

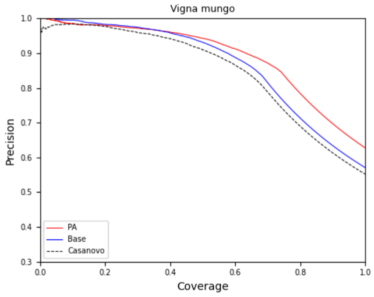

(i) *Vigna mungo*

Figure S3. **Peptide precision vs. coverage on the nine-species V1 dataset, training on 8 species and evaluating on the held-out 9th.** All individual species are displayed.

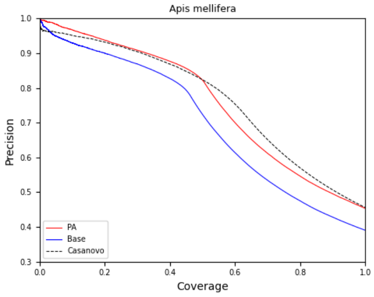

(a) *Apis mellifera*

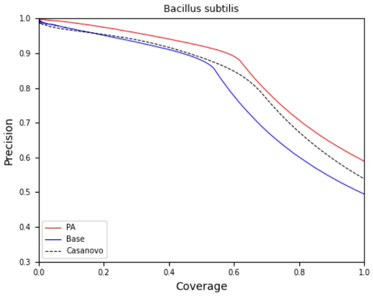

(b) *Bacillus subtilis*

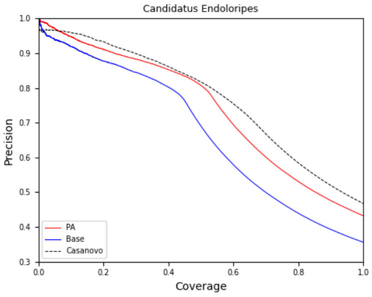

(c) *Candidatus endoloripes*

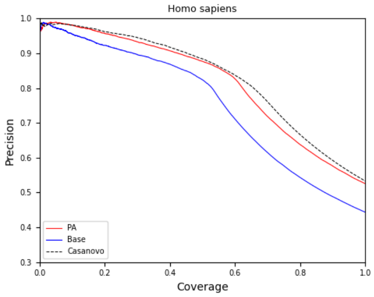

(d) *Homo sapiens*

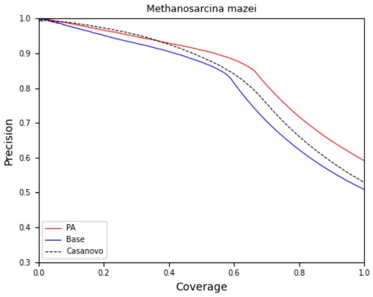

(e) *Methanosarcina mazei*

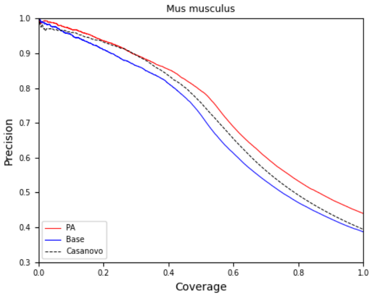

(f) *Mus musculus*

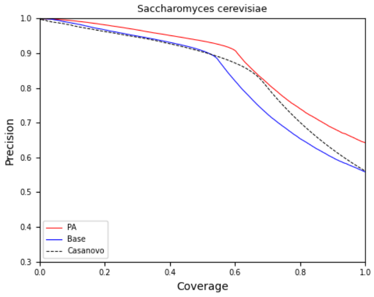

(g) *Saccharomyces cerevisiae*

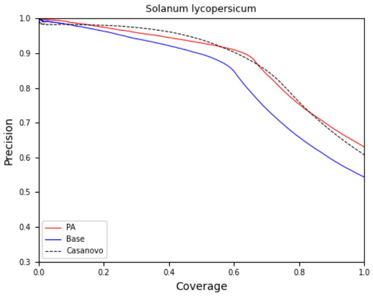

(h) *Solanum lycopersicum*

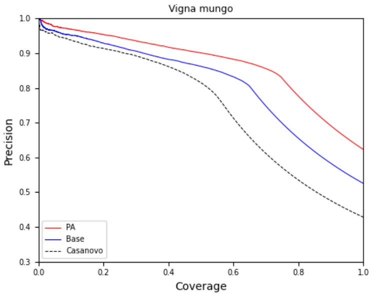

(i) *Vigna mungo*

Figure S4. **Peptide precision vs. coverage on the nine-species V2 dataset, training on 8 species and evaluating on the held-out 9th.** All individual species are displayed.

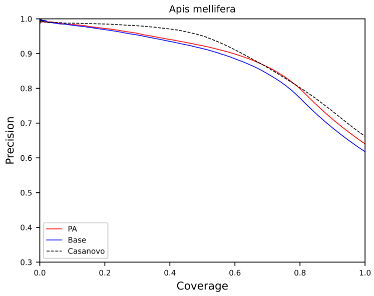

(a) *Apis mellifera*

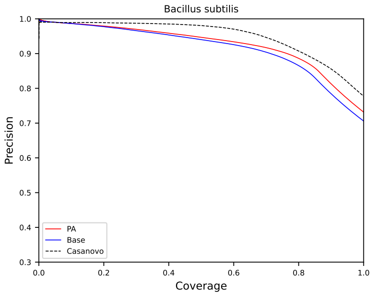

(b) *Bacillus subtilis*

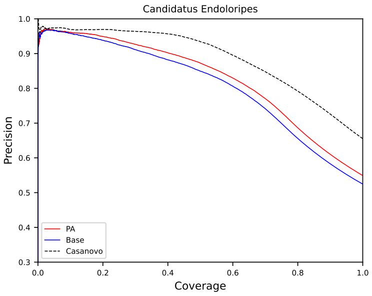

(c) *Candidatus endoloripes*

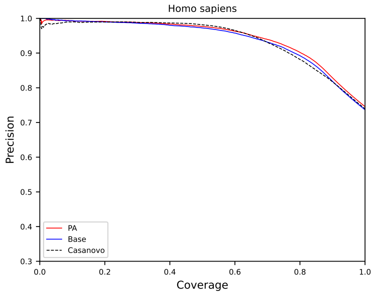

(d) *Homo sapiens*

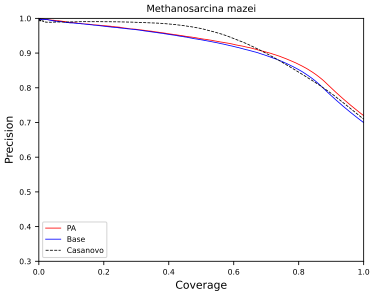

(e) *Methanosarcina mazei*

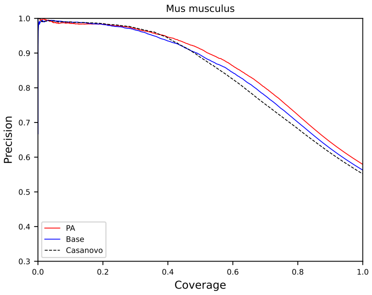

(f) *Mus musculus*

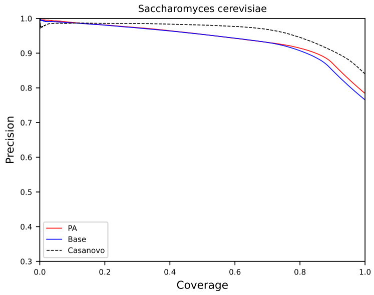

(g) *Saccharomyces cerevisiae*

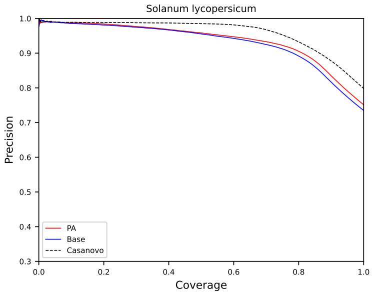

(h) *Solanum lycopersicum*

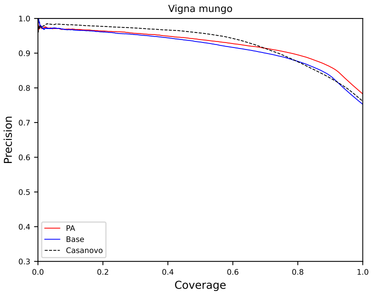

(i) *Vigna mungo*

Figure S5. **Peptide precision vs. coverage on the nine-species V2 dataset after training on the MassIVE-KB dataset.** All individual species results are shown separately.
